# Reviewer acknowledgement 2013

**DOI:** 10.1186/1471-2458-14-81

**Published:** 2014-02-21

**Authors:** Natalie Pafitis

**Affiliations:** 1 BioMed Central, 236 Gray’s Inn Road, London WC1X 8HB, UK

## Abstract

**Contributing reviewers:**

The editors of BMC Public Health would like to thank all our reviewers who have contributed to the journal in Volume 13 (2013).

## 

Leif Edvard A Aaroe

Norway

Shazia Abbas

Pakistan

Anees Ahmed Abdul Pari

UK

Sultana Abdulaziz

Saudi Arabia

Mohamed Hatha Abdulla

India

Asnawi Abdullah

Indonesia

Sawsan Abdulrahim

Lebanon

Fantu Abebe

Ethiopia

Gemeda Abebe

Ethiopia

Gillian Abel

New Zealand

Johan Abildgaard

Denmark

Seye Abimbola

Nigeria

Femke Abma

Netherlands

Raymond Aborigo

Ghana

Rachel T. Abraham

USA

Sandra Abreu

Portugal

Clemente Abrokwaa

USA

Sandro Accorsi

Italy

Jean Adams

UK

Madeleine Adams

Australia

Cheryl Addy

Qatar

Ayo Adebowale

Nigeria

Adedeji Adefuye

USA

Oluwatoyosi Adekeye

USA

Sunday Adedeji Aderibigbe

Nigeria

Ramesh Adhikari

Nepal

Shiva Adhikari

Nepal

Armita Adily

Australia

Visseho Adjiwanou

South Africa

Nancy Adler

USA

Richard Adome

Uganda

Maureen Adudans

Kenya

Wichai Aekplakorn

Thailand

Patricia Agaba

Nigeria

Suneth Agampodi

Sri Lanka

Thilini Chanchala Agampodi

Sri Lanka

Girdhar Agarwal

India

Keren Agay-Shay

Spain

Oche Agbaji

Nigeria

DeBrenna Agbenyiga

USA

Gabriel Agboado

UK

Sohail Agha

USA

Paul Agius

Australia

Charles Agyemang

Netherlands

Richard Ahlström

Sweden

Sk. Akhtar Ahmad

Bangladesh

Brian Ahmedani

USA

Jun Aida

UK

Olusegun Ajenifuja

Nigeria

Ademola Ajuwon

Nigeria

Saeed Akhtar

Kuwait

Noori Akhtar-Danesh

Canada

Olagoke Akintola

South Africa

Tomi Akinyemiju

USA

mohammed Al Ghobain

Saudi Arabia

Khuder Alagha

France

Arsham Alamian

USA

Ahamed Al-Amin

Sudan

Leena Ala-Mursula

Finland

David A. Albert

USA

Daniel Aldrich

USA

Sami Al-Dubai

Malaysia

Jolanta Aleksejuniene

Canada

Farrokh Alemi

USA

Neal Alexander

UK

Evangelos Alexopoulos

Greece

Nadia Ali

USA

Alaa Alkerwi

Luxembourg

Naomi Allen

UK

Amel Al-Makoshi

UK

Marie Alricsson

Sweden

Shirina Alsowaidi

United Arab Emirates

Ayman Alsulaiman

Saudi Arabia

Christian Althaus

Switzerland

Benjamin Althouse

USA

Laura C Altobelli

Peru

Bülent Altun

Turkey

Fátima Alves

Portugal

Alemayehu Amberbir

Malawi

Andrea Amici

Italy

Daniela Amicizia

Italy

Tarek Amin

Saudi Arabia

Olukemi Amodu

Nigeria

Amanda Amos

UK

Agbessi Amouzou

USA

Olubusola Amu

UK

Fotios Anagnostopoulos

Greece

Katherine Anders

Vietnam

Anette Andersen

Denmark

Brit Anderson

USA

Eleanor Anderson

UK

Ian Anderson

Austria

John Anderson

USA

Francis-Xavier Andoh-Adjei

Ghana

Flavia Andrade

USA

Laura Helena Andrade

Brazil

Stefan Andreas

Germany

Solange Andreoni

Brazil

Elena Andresen

USA

Floro Andrés-Rodríguez

Spain

Nadine Andrew

Australia

Aranka Anema

Canada

Johannes R Anema

Netherlands

Luis Anibarro

Spain

Daniel Ankrah

Ghana

Matilda Annerstedt

Sweden

Donna Ansara

USA

Y Gavriel Ansara

Australia

Rashid Ansari

Australia

Aderaw Anteneh

Ethiopia

Peter Anthamatten

USA

Georgios Antonogeorgos

Greece

William Anyan

Ghana

Virginia Aparicio

Spain

Carlos M Arango

USA

Chrisa Arcan

USA

Edward Archer

USA

Norm Archer

Canada

Daniel Argaw Dagne

Switzerland

Caroline Ariën

Belgium

Hisatomi Arima

Australia

Koya Ariyoshi

Japan

Anna Arlinghaus

Germany

Miranda Elaine Glynis Armstrong

UK

Benjamin Arnold

USA

Arpo Aromaa

Finland

Joanne Arsenault

USA

I Wayan Gede Artawan Eka Putra

Indonesia

Guilherme Artioli

Brazil

Oyedunni Arulogun

Nigeria

Daniel Arvidsson

Sweden

Lisa Aschan

UK

Pablo Aschner

Colombia

Tigistu Adamu Ashengo

USA

Kristin Ashford

USA

Stephen Asiimwe

Uganda

Ali Assabri

Yemen

Erika Elizabeth Atienzo

Mexico

Andrew J Atkin

UK

Robert Atkins

USA

Jo-An Atkinson

Australia

Evan Atlantis

Australia

Pierre Aubas

France

Lana Augustincic Polec

Canada

Dagfinn Aune

UK

Robert Aunger

UK

Marlein Ausems

Netherlands

Niyi Awofeso

Australia

Kofi Awusabo-Asare

Ghana

Kazi Md. Abul Kalam Azad

Bangladesh

Mohammad Azam

Australia

Kenichi Azuma

Japan

Ebenezer Baba

Nigeria

Olusegun Babaniyi

Zambia

Olusegun Babaniyi

Zambia

Juliet Babirye

Uganda

Kathryn Backholer

Australia

Kristina Bacon

USA

Hanan Badr

Kuwait

Alia Badri

Netherlands

Sunil Badve

Australia

Omar Bagasra

USA

Peter Baguma

Uganda

Luciana Bahia

Brazil

Mike Bailey

USA

Lisa Bailey-Davis

USA

Janette Baird

USA

Janis Baird

UK

John Kevin Baird

Indonesia

Anna Bajer

Poland

Dean Baker

USA

Kelly Balistreri

USA

Clare Bambra

UK

Emily Banks

Australia

Pam Banner

Australia

Sayera Banu

Bangladesh

Wei Bao

USA

Christopher Barbara

Malta

Lindley Barbee

USA

Maria Barcelo

Spain

Ilaria Barchetta

Italy

Alicia Barrasa

Spain

Tiago Barreira

USA

Emily Barrett

USA

Mel Bartley

UK

Jo Barton

UK

Iman Basheti

Jordan

Abdul Basit

Pakistan

Miia Bask

Norway

Tibor Baska

Slovakia

Eric Bateman

South Africa

Temesgen Bati

Ethiopia

Michael Batz

USA

Georg F. Bauer

Switzerland

Gerrit Bauer

Germany

Leah Bauer

USA

Himmatrao Bawaskar

India

Suzanne Domel Baxter

USA

Kaleab Baye

Ethiopia

Ana Baylin

USA

Alison Beauchamp

Australia

Roxanne Beauclair

South Africa

Annette Beautrais

USA

Eddy Beck

Switzerland

François Beck

France

Małgorzata Bednarska

Poland

Marin Been

Netherlands

Deborah Begoray

Canada

Donatien Beguy

Kenya

Sadjia Bekal

Canada

Joshua Bell

UK

Lucy Bell

Australia

Nick Bellissimo

Canada

Folasade Bello

Nigeria

Gianni Bellomo

Italy

Daniel Belsky

USA

Eugenio Beltran

USA

Ahmed Ben Abdelaziz

Tunisia

Adam Bennett

USA

Cecile Bensimon

Canada

Jacob Bentley

USA

Gláucia Benute

Brazil

Andrea Beratarrechea

Argentina

Janneke Berecki-Gisolf

Australia

Rigmor Berg

Norway

Yemane Berhane

Ethiopia

James Berkley

Kenya

Anita Berlin

Sweden

Anne H Berman

Sweden

Paquito Bernard

France

Kimberly Bernosky-Smith

USA

Ricardo Bertolla

Brazil

Pascal Bessong

South Africa

Jeffrey Bethel

USA

Katherine Bevans

USA

Abebe Beyene

Ethiopia

Nulda Beyers

South Africa

Caryl M Beynon

UK

Kavi Bhalla

USA

Ramesh Y. Bhat

India

Parinita Bhattacharjee

Kenya

Maryka H Bhattacharyya

USA

Gouri Sankar Bhunia

India

Zulfiqar Bhutta

Pakistan

Haim Bibi

Israel

Warren Bickel

USA

Damir Bikmukhametov

Netherlands

Gemma Binefa

Spain

Marco Biolato

Italy

Samuel Biraro

Uganda

Chris Birch

Australia

Wendy Birmingham

USA

Anikó Bíró

UK

Karen Bissell

New Zealand

Alessandra Bitto

Italy

Goran Bjelakovic

Serbia

Jonas Björk

Sweden

Emma Bjorkenstam

Sweden

Gunnar Aksel Bjune

Norway

Nathan Blanchet

USA

Patricia Blank

Switzerland

Steven Block

USA

Johannes Blom

Sweden

Ian Boardley

UK

Helen Boardman

UK

Martin Bobak

UK

Tomasz Bochenek

Poland

Nansi Boghossian

USA

Thomas Böhler

Germany

Catherine Bolman

Netherlands

Omotayo Bolu

USA

Andrea Bombak

Canada

Sebastien Bommart

France

Dale Bond

USA

Susan Bondy

Canada

Marcelo Bönecker

Brazil

Sandra Bonellie

UK

Sadie Boniface

UK

Celine Bonnet

France

Cecile RL Boot

Netherlands

Alison Booth

Australia

Robert Booth

USA

Michael Borg

Malta

Ron Borland

Australia

Luisa Borrell

USA

Sonia Borrell

Switzerland

Matthys (Hennie) Botha

South Africa

Pascal Bovet

Switzerland

Katherine Bowers

USA

Aaron Bowman

USA

Javier Boyas

USA

Allison Boyes

Australia

Loretta Brabin

UK

Kristina Brache

Canada

Robert Bradley

USA

Pamela Bradshaw

Australia

Lutgart Braeckman

Belgium

Nicola Luigi Bragazzi

Italy

Peter Branney

UK

Leah Brennan

Australia

Penny Brennan

USA

Joshua Breslau

USA

Cinzia Bressi

Italy

Thomas Brett

Australia

Serge Briançon

France

William Brieger

USA

Stephanie Brien

Canada

Frederic Briere

Canada

Julie Brimblecombe

Australia

Thomas Britt

Vanuatu

Paul Brocklehurst

UK

Robert Brooks

Australia

Hélène Broutin

France

Graham Brown

Australia

Heather Brown

UK

Helen Brown

Australia

Helen Elizabeth Brown

UK

Jamie Brown

UK

Joe Brown

UK

Jessica Browne

Australia

Mariana Brussoni

Canada

Stirling Bryan

Canada

Petra Brysiewicz

South Africa

Patrick Brzoska

Germany

Tania Bubela

Canada

William Buchta

USA

Jennifer Buher Kane

USA

Goof Buijs

Netherlands

Hilde Buiting

Netherlands

Alessandra Buja

Italy

Mohammed Bukar

Nigeria

Susan Bull

UK

David Buller

USA

Monika Bullinger

Germany

Alison Bullock

UK

Doina Bunescu

Romania

Emily Burger

Norway

Jeff Burgess

USA

Sara Burke

USA

Nicola Burton

Australia

Suzan Burton

Australia

Kathryn Bush

Canada

Valérie Buthion

France

India Butler

South Africa

Judith Buttriss

UK

Jane Buxton

Canada

Alberto Caban-Martinez

USA

Noriko Cable

UK

Cigdem Caglayan

Turkey

Xiaodong Cai

USA

Loretta Cain

USA

Lilian Calderon-Garciduenas

USA

Sarah Caldwell

UK

Russell Callaghan

Canada

Denton Callander

Australia

Geoffrey Calvert

USA

Vitaliano Cama

USA

Donatella Camerino

Italy

Adrian Cameron

Australia

Vicky Cameron

New Zealand

Ann Campagna

USA

Fiona Campbell

UK

Mayilee Canizares

Canada

Linda Cantley

USA

Jason Cao

USA

Martin Caraher

UK

Alex Carballo-Dieguez

USA

Nick Carcioppolo

USA

Hugo Cardoso

Portugal

Rachel Carey

Australia

Asa Carlsund

Sweden

Christin Carotta

USA

Catherine Carpenter

USA

L Rand Carpenter

USA

Peter Carrasco

USA

John Carstensen

Sweden

Owen Carter

Australia

Alison Carver

Australia

patrick Cashman

Australia

Susan Cassels

USA

Brenda Cassidy

USA

Mike Cassidy

Canada

Carlos Castañeda-Orjuela

Colombia

Gianluca Castelnuovo

Italy

Jesús Castilla

Spain

Fabian Cataldo

UK

Lucrezia Catania

Italy

Dolores Catelan

Italy

Julien Caudeville

France

Erica Cavanaugh

USA

Joan Cayla

Spain

Françoise Cazein

France

Adriana Ceci

Italy

Gabriel Chamie

USA

Supat Chamnanchanunt

Thailand

Juliana Chan

China

Lily Chan

Australia

Philip Chan

USA

Amar K Chandra

India

Abhirosh Chandran

Canada

Beth Chaney

USA

David Chaney

UK

Hung-Hao Chang

Taiwan

Jongwha Chang

USA

Mei-Wei Chang

USA

Dennis Chao

USA

Jianqian Chao

China

M Pia Chaparro

Sweden

Karen Chapman-Novakofski

USA

François Chappuis

Switzerland

Jean-Philippe Chaput

Canada

Arundhati Char

India

Abdurrahman Charkazi

Iran

Mutemba Charles

Zambia

Karen Charlton

Australia

Man Charurat

USA

Nearkasen Chau

France

Ken Checinski

UK

Patrick Chege

Kenya

Chunming Chen

China

Feng Chen

China

Jiaxu Chen

China

Hui Cheng

China

Jin-quan Cheng

China

John Cherrie

UK

Bernard Cheung

Hong Kong

Rosy Chhabra

USA

Hsin Chi

Taiwan

Carlos Chiatti

Italy

Dixon Chibanda

Zimbabwe

Carla Chibwesha

Zambia

Jephat Chifamba

Zimbabwe

Palma Chillón

Spain

Katharine Chisholm

UK

Mohammod Chisti

Bangladesh

Angela Chiu

USA

Po-Ju Chiu

Taiwan

Lillian Choi

USA

Herberto Jose Chong Neto

Brazil

Kee-Lee Chou

China

Patricia Chou

USA

CB Chow

Hong Kong

Karl Bang Christensen

Denmark

Deborah Christie

UK

Nicola Christie

UK

Christina Chrysohoou

Greece

JongSerl Chun

South Korea

Manuel Cifuentes

USA

Donna Ciliska

Canada

Arzum Ciloglu

USA

Caterina Cinel

UK

Cheryl Clark

USA

Antonio Clavenna

Italy

Els Clays

Belgium

Tom Clemens

UK

Stacy Clemes

UK

Mario Clerici

Italy

Caroline Cobb

USA

Renata Cocco

Brazil

Anne Cockcroft

Botswana

Fiona Cocker

Australia

Patricia Coffey

USA

David Coggon

UK

Alison Cohen

USA

Arnon Cohen

Israel

Vittoria Colamesta

Italy

Donn Colby

Viet Nam

Sarah Colby

USA

Matthew Coldiron

France

Donald Cole

Canada

Samantha Cole

USA

Brenda Coleman

Canada

Paul Collings

UK

Martine Collumbien

UK

Elien Colman

Belgium

Katherine Conigrave

Australia

Rudi Coninx

Switzerland

Francesca Conradie

South Africa

Sharon Conroy

UK

Rena Conti

USA

Paul Maurice Conway

Denmark

Adrian Cook

UK

Angus Cook

Australia

Jackie Cook

Tanzania

Mary Cooke

UK

Kerri Coomber

Australia

Emma Coombes

UK

Keith Cooper

UK

Erwin Cooreman

Myanmar

Kirsten Coppell

New Zealand

Kirsten Corder

UK

Inge Corless

USA

Heather Corliss

USA

Patrícia Corrêa-Faria

Brazil

Sofia Correia

Portugal

Daniel J Corsi

USA

Giuliana Cortese

Italy

Marco Cosentino

Italy

Silvia Costa

UK

Lesley Cottrell

USA

Ryan Courtney

Australia

Frances Cowan

UK

Georgina Cox

Australia

Peter Craig

UK

Susanna Cramb

Australia

Paul Crawshaw

UK

Georgina Crichton

Australia

Christine Critchley

Australia

Peter Croft

UK

Mathilde Crone

Netherlands

Wendy Cronin

USA

Kenny Crump

USA

Rik Crutzen

Netherlands

Magdalena Cuenca Garcia

Spain

Diana Cunha

Portugal

Cheryl Currie

Canada

Marian Currie

Australia

Andrew Curtis

UK

Peter D’abbs

Australia

Stephan Dahl

UK

Qi Dai

USA

Magbagbeola David Dairo

Nigeria

Elias Dakwar

USA

Elvira D’Andrea

Italy

Stefania D’Angelo

UK

Anna-Karin Danielsson

Sweden

Nadia Danon-Hersch

Switzerland

Ina Danquah

Germany

Andrew Darnton

UK

Jishnu Das

USA

Mike Daube

Australia

Luc Dauchet

France

AuTumn Davidson

USA

Patricia Davidson

Australia

Gabrielle Davie

New Zealand

Kia Davis

USA

Sally M Davis

USA

Gareth Davison

UK

Symeon Davkas

UK

Brenda Davy

USA

Jennifer Day

UK

Sajal De

India

Jessica de Beer

Netherlands

Freia De Bock

Germany

Bart De Clercq

Belgium

Katrien De Cocker

Belgium

Ayesha De Costa

Sweden

Maximilian de Courten

Denmark

Ellen De Decker

Belgium

Augusto Cesar de Moraes

Brazil

Jascha de Nooijer

Netherlands

Ellen De Pauw

Belgium

Karin A De Ridder

Belgium

Angelique de Rijk

Netherlands

Mary De Silva

UK

Henrica de Vet

Netherlands

Gerard de Vries

Netherlands

Chiara de Waure

Italy

Leonore M. de Wit

Netherlands

Kyle De Young

USA

Wesley Dean

USA

Kevin Deane

USA

Raisa Deber

Canada

Jessika Deblonde

Belgium

Lilliana Del Busso

Norway

Louise Delany

New Zealand

Sinead Delany

South Africa

Barbara Deller

USA

Paolo Deluca

UK

Allard Dembe

USA

Anahit Demirchyan

Armenia

Zewditu Demissie

USA

Pascal Demoly

France

Margriet den Boer

UK

Brenda Den Oudsten

Netherlands

Dwight Denison

USA

Rodolfo Dennis

Colombia

Lynette Denny

South Africa

Licia Denti

Italy

Cheryl Der Ananian

USA

Assefa Seme Deresse

Ethiopia

Kebede Deribe

Ethiopia

Laurie DeRose

USA

Angelo d’Errico

Italy

Sarika Desai

UK

Jean-Claude Desenclos

France

Loic Desquilbet

France

Muluken Dessalegn

Ethiopia

Roger Detels

USA

Isabelle Devaux

Sweden

Sarah Dewing

South Africa

Vidyasagar Dharmapuri

USA

Mukesh Dherani

UK

Nathalie Dhont

Belgium

Aldo Di Carlo

Italy

Ross Di Corleto

Australia

James Dickinson

Canada

Kacie Dickinson

Australia

Petra Dickmann

UK

Triantafillos Didangelos

Greece

Patricia Dietz

USA

Joseph DiFranza

USA

Henriëtte Dijkshoorn

Netherlands

Peggye Dilworth-Anderson

USA

Nedialko Dimitrov

USA

Helen Ding

USA

Yingying Ding

China

Rylee Dionigi

Australia

Vallop Ditsuwan

Thailand

Fahdi Dkhimi

Belgium

Khoi Do

Australia

Young Kyung Do

Singapore

Bahar Dogan

Turkey

Nurhan Dogan

Turkey

Mirriam Dogimab

Papua New Guinea

Philip Domeyer

Greece

Emma Donaldson-Feilder

UK

Xiao Dong

USA

Amol Dongre

India

Kyla Donnelly

USA

Rob Donovan

Australia

Shannon Doocy

USA

Moira Doolan

UK

Gregory Dore

USA

Geme Urge Dori

Ethiopia

Maria Dorrucci

Italy

Solange dos Santos

Brazil

Sucheta Doshi

USA

Michael Doumas

Greece

Jacqueline Doumit

Lebanon

Orla Doyle

Ireland

Robert Dreibelbis

USA

Maren Dreier

Germany

Jens Dreyhaupt

Germany

Melanie Drolet

Canada

David Dror

India

Li-Ping Duan

China

Martin Duerden

UK

Kiyah Duffey

USA

Marylene Dugas

Canada

Pierre Dujols

France

Dora Dumont

USA

Julien Dumurgier

France

Dustin Duncan

USA

Myanna Duncan

UK

Ruth Dundas

UK

Elizabeth Dunford

Australia

Jean Dupouy-Camet

France

David Durham

USA

Anne Durkan

Australia

Ambarish Dutta

India

Rina Dutta

UK

David Dzewaltowski

USA

Valerie Earnshaw

USA

Lisa Eaton

USA

Victoria Edge

Canada

Katie Edwards

USA

Maria A Efstratiou

Greece

Katie Egan

USA

Grace Egeland

Norway

Thorlene Egerton

Norway

Thaddaeus Egondi

Kenya

Andrey Egorov

Germany

Deborah Ehrenthal

USA

Stephan Ehrhardt

USA

Rochelle Eime

Australia

Goran Ejlertsson

Sweden

Lilani Ekanayake

Sri Lanka

Björn Ekman

Sweden

Robert Ekman

Sweden

Uwem Ekpo

Nigeria

Karima El Rhazi

Morocco

Tobias Elgan

Sweden

Carol El-Hayek

Australia

Thomas Elkeles

Germany

Ziad El-Khatib

Sweden

Raina Elley

New Zealand

Michael Elliott

USA

Philippa Ellwood

New Zealand

Roberto Elosua

Spain

Peter Elwood

UK

Saad Elzanaty

Sweden

Pauline Emmett

UK

Ingunn Engebretsen

Norway

Lina Engelen

Australia

Vikki Entwistle

UK

Rajiv Erasmus

South Africa

Gizem Erdem

USA

Charli Eriksson

Sweden

Temitope Erinosho

USA

Ozum Erkin Balyaci

Turkey

Susan Ernst

USA

Johanna Eronen

Finland

Jenni Ervasti

Finland

Jorge Escobedo

Mexico

Cam Escoffery

USA

Reuben Escorpizo

Switzerland

Abdulrazaq Esin

Nigeria

Laura Esmail

USA

Claudia Estcourt

UK

Belachew Etana

Ethiopia

Elizabeth Evans

UK

Sherrill Evans

UK

William Evans

USA

Rebecca Evans-Polce

USA

Natalie Exner

USA

Deinera Exner-Cortens

USA

Helen Eyles

New Zealand

Ifeanyichukwu Ezebialu

Nigeria

Kate Faasse

New Zealand

William Fabricius

USA

Lars Thore Fadnes

Norway

David Faeh

Switzerland

Lavinia Falese

Italy

Amanda Fallin

USA

Li-Qun Fang

China

José Cazuza Farias Júnior

Brazil

Anna Farmer

Canada

Richard Farmer

USA

Stephen Farrall

UK

Konstantinos Farsalinos

Greece

Margaret Fatudimu

Nigeria

Giampiero Favato

UK

Olufunmilayo Fawole

Nigeria

Michael Fay

USA

Ousmane Faye

Senegal

Bruno Federico

Italy

Marcus Feldman

USA

Eleonora Feletto

Australia

Cindy Feng

Canada

Mogens Fenger

Denmark

Sara Ferlander

Sweden

Meenakshi Fernandes

Italy

Romulo Fernandes

Brazil

Esteve Fernandez

Spain

Frans J.M. Feron

Netherlands

Marco Mario Ferrario

Italy

Jane Ferrie

UK

Edith Feskens

Netherlands

James Fielding

Australia

Richard Fielding

Hong Kong

Albert Figueras

Spain

Filippos Filippidis

Greece

Sara Filoche

New Zealand

Gunther Fink

USA

Eric Finkelstein

Singapore

Sarah Finocchario Kessler

USA

Katherine Fiori

USA

Jeremy Firestone

USA

Frida Fischer

Brazil

Colleen Fisher

USA

Helen Fisher

UK

Brendan Flannery

Brazil

Elaine Fleck

USA

Jennifer Flegg

UK

Fiona Fleming

UK

Steffen Flessa

Germany

Benjamin Fletcher

UK

Nils Fleten

Norway

Ingrid Flight

Australia

Ewa Florek

Poland

Stephen Flores

USA

Marco Floridia

Italy

Alex Florindo

Brazil

Paul Flowers

UK

Erin Flynn-Evans

USA

George Fodor

Canada

Louise Foley

UK

Perry Foley

USA

Maria Jo&atilde;o Fonseca

Portugal

Maria João Fonseca

Portugal

Kevin Fontaine

USA

Anthony Fooks

UK

Francesco Forastiere

Italy

Laura Forbes

Canada

Kelsie Forbush

USA

Dara Ford

USA

Earl Ford

USA

Ann Forsyth

USA

Sandrine Fosse-Edorh

France

John Foster

UK

Jean Christophe Fotso

Kenya

Ashley Fowlkes

USA

Philippe Fraisse

France

Christina Frank

Germany

Eleonor Fransson

Sweden

Paulo Frazao

Brazil

Becky Freeman

Australia

Matthew Freeman

USA

Georgia Frey

USA

Ulrich Frick

Austria

Christine Friedenreich

Canada

Alexander W. Friedrich

Netherlands

Sharon Friel

Australia

Sari Fröjd

Finland

Chaowei Fu

China

Ping Fu

China

Steven Fu

USA

Adrian Fuente

Australia

Yoshikazu Fujisawa

Japan

Yoshinori Fujiwara

Japan

Bo Fusek

Canada

Knut Fylkesnes

Norway

John Gabbay

UK

Belinda Gabbe

Australia

Roman Gabrhelik

Czech Republic

Erica Gadsby

UK

Jacques Gaillat

France

Sally Gainsbury

Australia

Maria Rosaria Galanti

Sweden

Alison Gallagher

UK

Danielle Gallegos

Australia

Silvano Gallus

Italy

Elvis Gama

UK

John Gamble

USA

Anne Sophie Gamez Dubuis

France

Rashmi Gangamma

USA

Stuart Gansky

USA

Marie Gantz

USA

Dana Garbarski

USA

Maria Luiza Garcia Rosa

Brazil

Olaya García-Rodríguez

Spain

Benjamin Gardner

UK

Genevieve Gariepy

Canada

Susan Garrett

New Zealand

Didier Garriguet

Canada

Fania Gärtner

Netherlands

Roberto Gasparini

Italy

Peter Gaturuku

Congo

Alain Gauthier

Canada

Rukhsana Gazi

Bangladesh

Andrea Gazzinelli

Brazil

Alan Geater

Thailand

Haftu Berhe Gebru

Ethiopia

Lot Geels

Netherlands

Scott Geibel

Zambia

Becky Genberg

USA

Joan Gene Badia

Spain

Emma George

Australia

Oommen George

India

Patricia Gerakopoulou

Greece

Sanne Gerards

Netherlands

Mulusew Gerbaba

Ethiopia

Markus Gerber

Switzerland

Danielle German

USA

Francesco Gesualdo

Italy

Fentie Getahun

Ethiopia

Huda Gharaibeh

Jordan

Mojgan Gharipour

Iran

Musie Ghebremichael

USA

Rohini Ghosh

India

Sanjob Ghosh

India

Fabrizio Giannandrea

Italy

Graham Gibbs

Canada

Mahée Gilbert-Ouimet

Canada

Melissa Gilkey

USA

Simone Gill

USA

Melissa Gilliam

USA

Hans Gilljam

Sweden

Chris Gingrich

USA

Guglielmo Giraldi

Italy

Dorota Girard

France

Rosa gispert

Spain

Anna Giuliano

USA

Gerd M. Glaeske

Germany

Garrett Glasgow

USA

Deborah Glik

USA

Roberto Gnavi

Italy

Wendy Gnich

UK

Tesfaye Gobena

Ethiopia

Dionne Godette

USA

Shifalika Goenka

India

Judy Gold

Australia

Lisa Gold

Australia

Daniel Goldberg

USA

Shira Goldenberg

USA

Gabriela B Gomez

Netherlands

Alejandro Gómez Bruton

Spain

Javier Gomez de Terreros

Spain

Alba Gomez-Cabello

Spain

Jorge-Enrique Gomez-Marin

Colombia

Marta Gonçalves

Portugal

Juan Marcos Gonzalez

USA

Vivian Gonzalez

USA

Alejandro González-Agüero

Spain

Marcela Gonzalez-Gross

Spain

Arturo Gonzalez-Izquierdo

UK

Holly Gooding

USA

Patrick Goodman

Ireland

Denise Goodwin

UK

Pierre Goovaerts

USA

Ross Gordon

Australia

Trish Gorely

UK

Giuseppe Gorini

Italy

George Gotsadze

Georgia

Agnes Gozdzik

Canada

Luis Gracia-Marco

UK

Ana Soledade Graeff-Martins

Brazil

Petra Graham

Australia

Rebecca Grais

France

Bibi Gram

Denmark

Rachel Grana

USA

Michael Grandner

USA

Gerlinde Grasser

Austria

Cindy Gray

UK

Joanne Gray

UK

Stuart Gray

UK

Michael James Green

UK

Trisha Greenhalgh

UK

Katie Greenland

UK

Amy Greer

Canada

Simon Gregson

UK

Christine Grella

USA

Francis Grenier

Japan

Gloria Grice

USA

Jessica Grieger

Australia

Jeffrey Grierson

Australia

Andrej M Grjibovski

Norway

Danielle Groffen

Netherlands

Sareen Gropper

USA

Christian Grov

USA

Klaus G. Grunert

Denmark

Thomas Guadamuz

Thailand

Maria Rosaria Gualano

Italy

Richard Guerrant

USA

Fernando Guerrero-Romero

Mexico

Debby Guha-Sapir

Belgium

Martin Guhn

Canada

Shanna Guilfoyle

USA

Fernando Guilherme da Costa

Brazil

Martin Gulliford

UK

Freedom Nkhululeko Gumedze

South Africa

Craig Gundersen

USA

Fangjian Guo

USA

Hongbin Guo

Canada

Hongxiong Guo

China

How-Ran Guo

Taiwan

Yan Guo

China

Digant Gupta

USA

Sanjeev Gupta

India

Anup Gurung

Papua New Guinea

Alison Gustafson

USA

Jillian Guthrie

Australia

Juan Pablo Gutierrez

Mexico

Steffany Haaz

USA

Jessica Haberer

USA

Rima Habib

Lebanon

Mariana Hacker

Brazil

Beverley Haddad

South Africa

Slim Haddad

Canada

Christopher Haddock

USA

James Hadler

USA

Patricia Hageman

USA

Gareth Hagger-Johnson

UK

Fahimeh Haghighatdoost

Iran

Akiko Hagimoto

Japan

Bridget Haire

Australia

Peter Hajek

UK

Mohammad Hajizadeh

Canada

Amal Halder

Bangladesh

Danielle Haley

USA

Brian Hall

China

Mats Hallgren

Sweden

Heikki Hämäläinen

Finland

Tonelle Handley

Australia

David Hanley

Canada

Peter Hannan

USA

Nathan Hansen

USA

Chun Hao

USA

Zaeem Haq

Pakistan

Sobia Haqqi

Pakistan

Katherine Hardcastle

UK

Jessica Harding

Australia

Louise Hardy

Australia

Amal Harrati

USA

Kathleen Harrington

USA

Kristina Harris

USA

Flo Harrison

UK

Ingunn Harstad

Norway

Graham Hart

UK

Jaime Hart

USA

Marieke Antonia Hartman

USA

William Hartmann

USA

Samuel Harvey

UK

Melissa Haswell

Australia

Stephani Hatch

UK

Annemien Haveman-Nies

Netherlands

Gail Hawkes

Australia

Alison Hayes

Australia

Na He

China

Sara Head

USA

Gregory Heath

USA

Matthew Heaton

USA

Emily Hebert

USA

Tove Hedenrud

Sweden

Britt Hedman Ahlström

Sweden

Jean-Luc Heeb

Switzerland

Yvonne Heerkens

Netherlands

Jane Heffernan

Canada

Ulrich Hegerl

Germany

Christine Heidebrecht

Canada

Wieke H. Heideman

Netherlands

Christin Heidemann

Germany

Katriina Heikkila

Finland

Robert Heimer

USA

Ilkka Heinonen

Netherlands

Katie Heinrich

USA

Asgeir R. Helgason

Sweden

Margaret Hellard

Australia

Stefanie Helmer

Germany

Max Henderson

UK

Yaakov Henkin

Israel

Denise Heppe

Netherlands

Michael Hermanussen

Germany

Carmen Hernández-Martínez

Spain

Stephen Herrmann

USA

Susan Herrmann

Australia

Jean Hertzberg

USA

Tamara Hervey

UK

Sereina Herzog

Austria

Kathryn Hesketh

UK

Isabel Hess

Australia

Sonja Hess

USA

Paul Hewson

UK

Nick Hex

UK

Peter Heywood

Australia

Katalin Hideghéty

Hungary

Robin Hieblinger

Germany

Mitsuru Higuchi

Japan

Marianne Hillemeier

USA

Janet Hiller

Australia

Frances Hillier-Brown

UK

David Himmelgreen

USA

Trina Hinkley

Australia

Yoichiro Hirakawa

Australia

John Hirdes

Canada

Raghavendra Hl

India

Erin Hobin

Canada

Rebecca Kate Hodder

Australia

Christian Hoebe

Netherlands

Janet Hoek

New Zealand

Danielle Hollar

USA

Jo Holliday

UK

Samantha Hollingworth

Australia

Gunilla Hollman Frisman

Sweden

Amelia Hollywood

UK

Gerd Holmboe-Ottesen

Norway

Bjørn E. Holstein

Denmark

Martin Holt

Australia

Martin Holzhausen

Germany

Rianne Honigh-de Vlaming

Netherlands

Jan Hontelez

Netherlands

Robert Borden Hopkins

Canada

Gönül Dinç Horasan

Turkey

Peter Horby

Viet Nam

Samar Hore

Bangladesh

Bernardo Horta

Brazil

Susan Horton

Canada

Pia Horvat

UK

Caroline Horwath

New Zealand

Akiko Hosler

USA

Toru Hosoda

Japan

Matthew Hotopf

UK

Dismand Houinato

Benin

Catherine Houlihan

UK

Rawleigh Howe

Ethiopia

Denise Howel

UK

Juan Hoyos

Spain

Yu-Hsiang Hsieh

USA

Chien-Ning Hsu

Taiwan

Chun-Hsien Hsu

Taiwan

Cheng Huang

USA

Chih-Ling Huang

Taiwan

Song Lih Huang

Taiwan

Sue Huang

New Zealand

Ya Jun Huang

Hong Kong

Anne Hublet

Belgium

Ben Hudson

New Zealand

Darrell Hudson

USA

David Huebner

USA

Lindsay Huffhines

USA

Clare Hughes

Australia

Joel Hughes

USA

Karen Hughes

UK

Marian Huhman

USA

Vanora Hundley

UK

Jennifer Hunter

USA

Ruth Hunter

UK

Yanying Huo

USA

Anke Huss

Switzerland

Rafat Hussain

Australia

Corinne Husten

USA

Judy Hutchings

UK

Lieven Huybregts

Belgium

Sel Hwahng

USA

Nahla Hwalla

Lebanon

Jongnam Hwang

Canada

Won Ju Hwang

South Korea

Jim Hyde

Australia

Martin Hyde

Sweden

Hassen Idrees

Ethiopia

Umar Ikram

Netherlands

Aamer Imdad

USA

Cristina Inclan Valadez

Mexico

Victor Inem

Nigeria

Ioannis Ioannidis

Greece

Giuseppe Ippolito

Italy

Masahiro Irie

Japan

Jennifer D. Irwin

Canada

David Isaacs

Australia

Petros Isaakidis

India

Md Islam

Canada

Leyla Ismayilova

USA

Till Ittermann

Germany

Coen J. Itz

Netherlands

Silvina Ivalo

Argentina

Chimaraoke Izugbara

Kenya

Anne Jääskeläinen

Finland

Stefan Jackowski

Canada

Jonathan Jackson

UK

Rod Jackson

New Zealand

Angela Jackson-Morris

UK

Cristina Jacob

Brazil

Dany Jaffuel

France

Jagnoor Jagnoor

Australia

Russell Jago

UK

Albrecht Jahn

Germany

Animesh Jain

India

Irena Jakopanec

Norway

Katherine James

USA

Paul James

Canada

Peter James

USA

Mohamed Janabi

Tanzania

Bati Jango

Ethiopia

Fanny Janssen

Netherlands

Inma jarrin

Spain

Pujol Jean Louis

France

William Jeffcoate

UK

Amy Jeffers

USA

Johan H Jendle

Sweden

Emily Jenkins

Canada

Garry Jennings

Australia

Chris Jensen

Denmark

Jan L Jensen

Canada

Ole Kudsk Jensen

Denmark

Charlotte Jeppesen

Denmark

Dar-Der Ji

Taiwan

Jianguang Ji

Sweden

Cun-Xian Jia

China

Wang Jiaji

China

Stephanie Jilcott Pitts

USA

Ana Isabel Jimenez

Spain

Rodrigo Jimenez Garcia

Spain

Silvia Jimenez-Jorge

Spain

David Jimenez-Pavon

Spain

David JImenez-Pavon

Spain

Jun Jing

China

Oystein Haarklau Johansen

Norway

Juergen John

Germany

Ann-Christin Johnson

Sweden

Daniel Johnson

USA

Fiona Johnson

UK

Joy Johnson

Canada

Susan Johnson

USA

Tammie Johnson

USA

Sharon Johnston

Canada

Vanessa Johnston

Australia

Alan Wayne Jones

Sweden

Amanda C. Jones

Canada

David Jones

USA

Jermaine Jones

USA

Kay Jones

Australia

Laura Jones

UK

Tiffany Jones

Australia

József Gábor Joó

Hungary

Katja Joronen

Finland

KS Joseph

Canada

Malin Josephson

Sweden

Spruha Joshi

USA

Stephane Jouneau

France

Karina Julian

Argentina

Dolors Juvinya

Spain

Zubair Kabir

Ireland

Ephantus Kabiru

Kenya

Andrew. T Kaczynski

USA

Joseph Kagaayi

Uganda

Kai Kahl

Germany

Joseph Kaholokula

USA

Bernard Kaic

Croatia

Raj Kalaria

UK

Ramune Kalediene

Lithuania

Dorota Kaleta

Poland

Karrie Kalich

USA

Håkan Källmén

Switzerland

Ameeta Kalokhe

USA

Joan Kalyango

Uganda

Shaden Kamhawi

USA

Claire Kamp Dush

USA

Gagandeep Kang

India

Young Ae Kang

South Korea

Marko Kantomaa

Finland

Nilamadhab Kar

UK

Bulent Karadag

Turkey

Rozina Karmaliani

Pakistan

Taha Kass-Hout

USA

Saba Kassim

UK

Jodie Katon

USA

Dolores Katz

USA

Joanne Katz

USA

David Katzenstein

USA

Manas Kaushik

USA

Anne Kavanagh

Australia

Kim Kavanagh

UK

Maryam Kavousi

Netherlands

Yoko Kawaguchi

Japan

Elizabeth Kay

UK

Frances Kay-Lambkin

Australia

Aadil Kazi

UK

Wojciech Kazimierz

Poland

Michael Keall

New Zealand

Eimear Keane

Ireland

Patricia Kearney

Ireland

Catherine Keating

Australia

Aleksandar Kecojevic

USA

Swati Kedia Gupta

India

Tessa Keegel

Australia

Michelle Kegler

USA

Hunyinbo Kehinde Isaac

Nigeria

Mark Keim

USA

Roya Kelishadi

Iran

Brian Kelly

USA

Bridget Kelly

Australia

Cheryl Kelly

USA

Joel Kelso

Australia

Han Kemper

Netherlands

Andre Pascal Kengne

South Africa

Ryan David Kennedy

USA

Sheila Keogan

Ireland

Ruth Keogh

UK

Kiarri Kershaw

USA

Janko Kersnik

Slovenia

Joanna Kesten

UK

Naila Khalil

USA

Mohammad Khalili

Iran

Samoel Khamadi

Kenya

Asad Khan

Australia

Hassan Khan

UK

Mohammad Khan

South Korea

Rownak Khan

USA

Shusmita Hossain Khan

Bangladesh

Rasheda Khanam

Australia

Manaf Khatib

UK

Olga Khavjou

USA

Geok Lin Khor

Malaysia

Davoud Khorasani-Zavareh

Sweden

Davoud Khorasani-Zavareh

Iran

Sadik Khuder

USA

Susan M. Kiene

USA

Valerian Kiggundu

Uganda

Annice Kim

USA

Deok Ryun Kim

South Korea

Jaeyoun Kim

South Korea

Jaeyoung Kim

South Korea

Jane Kim

USA

Tadele Kinati

Ethiopia

Terry Kind

USA

Bill King

Australia

Carina King

UK

Daniel King

Australia

Malcolm King

Canada

Safari Kinung’hi

Tanzania

Susan Kippax

Australia

James Bowes Kirkbride

UK

Carmen Kirkness

USA

Litza Kiropoulos

Australia

Reiko Kishi

Japan

Ali Kitis

Turkey

France Kittel

Belgium

Pia Kivelä

Finland

Marc Kiviniemi

USA

Stephan Klasen

Germany

Jakob Klein

Austria

Zalika Klemenc-Ketis

Slovenia

Eveline Klinkenberg

Netherlands

Keith Klostermann

USA

Stephanie Knaak

Canada

Marit Knapstad

Norway

Lee Knifton

UK

Christina Knussen

UK

Ying-Chin Ko

Taiwan

Paul Kocken

Netherlands

Andreas Rembert Koczulla

Germany

steve koester

USA

Takehiko Koji

Japan

Anna Kokkevi

Greece

Lauri Kokkinen

Finland

Emmanuel Koku

USA

Gerasimos Kolaitis

Greece

Tracy Kolbe-Alexander

South Africa

Naoki Kondo

Japan

Kika Konstantinou

UK

Hanna Konttinen

Finland

Malcolm Koo

Taiwan

Katarzyna Kordas

USA

Rachel Kornfield

USA

Mette Korshøj

Denmark

Titia Kortbeek

Netherlands

Jan Kottner

Germany

Katharina Kovacs Burns

Canada

Joan Kowolik

USA

Digsu Negese Koye

Ethiopia

Augustine Kposowa

USA

Nana Kragh

Denmark

Douglas Krakower

USA

Ramona Krauss

USA

Anand Krishnan

India

Petter Kristensen

Norway

Jesper Krogh

Denmark

Katja Kröller

Germany

Hans Kromhout

Netherlands

Paul Krueger

Canada

Annamarie Kruger

South Africa

Gina Kruse

USA

Karolina Krysinska

Belgium

Violaine Kubiszewski

France

Chiharu Kubo

Japan

Koichiro Kudo

Japan

Sharon Kuhlmann

Sweden

Ramesh Kumar

Pakistan

Supriya Kumar

USA

Abera Kumie

Ethiopia

Goro Kuno

USA

Caroline Kuo

USA

Hannah Kuper

UK

Nyaradzai Edith Kurewa

Zimbabwe

Johanna Kuusisto

Finland

Craig Kuziemsky

Canada

Jerzy Kuzma

Papua New Guinea

Laura Kuznetsov

UK

Jan-Magnus Kvamme

Norway

Joseph Kvedar

USA

Zachary Kwena

Kenya

Wing Yun Kwok

Hong Kong

Jeff Kwong

Canada

Rurik L&ouml;fmark

Sweden

Florian Labhart

Switzerland

Luise Lago

Australia

Chandrakant Lahariya

India

Marjaana Lahti-Koski

Finland

Piero Luigi Lai

Italy

Rob Lake

New Zealand

Jeroen Lakerveld

Netherlands

PVM Lakshmi

India

Wendy Wing Tak Lam

Hong Kong

Karen Lamb

Australia

Anthony Daniel LaMontagne

Australia

Jonas Landberg

Sweden

Bodil Landstad

Sweden

Aoife Lane

Ireland

Katrin Lang

Estonia

Theis Lange

Denmark

Ricky Langley

USA

Mary Ann Lansang

Philippines

Victor Lara

Kenya

Ronaldo Laranjeira

Brazil

Linda Larcombe

Canada

Sarah Larney

Australia

Bernard Larouze

France

Bruce Larson

USA

Elysia Larson

USA

Matz Larsson

Sweden

Eva Lathrop

USA

Renat Latipov

Uzbekistan

Claudia Lau

USA

Gilbert Lau

Singapore

Jonathan Laugharne

Australia

Patrizia Laurenti

Italy

Francine Lavoie

Canada

Chi-kin Law

Hong Kong

Taiwo lawoyin

Nigeria

Lovett Lawson

Nigeria

Bradley Layton

USA

Lambros Lazuras

Greece

Lydie Le Mével

Switzerland

Nicole Le Saux

Canada

Toby Lea

Australia

Jennifer Leaning

USA

Justine Leavy

Australia

Dusica Lecic Tosevski

Serbia

Albert Lee

Hong Kong

Christina Lee

USA

Crystal Lee

Australia

Eun-Hee Lee

South Korea

Jie-Min Lee

Taiwan

Jong-Tae Lee

South Korea

Miryoung Lee

USA

Romeo Lee

Philippines

Wui-Chiang Lee

Taiwan

Yong-Jae Lee

South Korea

Yunhwan Lee

South Korea

Shelley Lees

UK

Thomas Lehnert

Germany

Catherine Lejeune

France

Stavroula Leka

UK

Zsuzsanna Lelovics

Hungary

Traci LeMasters

USA

Catherine Lemiere

Canada

Kathleen Lenk

USA

Alison Lennox

UK

Michela Lenzi

Italy

Katherine Lepani

Australia

Toby Leslie

UK

Richard Lester

Canada

William Letson

USA

Bianca Lever- van Milligen

Netherlands

Daphna Levinson

Israel

Cheryl Levitt

Canada

Glyn Lewis

UK

John Lewis

USA

Sophie Lewis

Australia

Els Leye

Belgium

Jian Li

Germany

Kaigang Li

USA

Leah Li

UK

Liming Li

China

Nanfang Li

China

Shenghui Li

China

Xinhu Li

China

Yang Li

China

Ying Li

China

Wee Liang En Ian

Singapore

Adrian Liau

USA

Ulrik Lidwall

Sweden

Arve Lie

Norway

Kelly Liebman

USA

Angela Liese

USA

Hyun-Sul Lim

North Korea

Yvonne Ai Lian Lim

Malaysia

Cheng-Chieh Lin

Taiwan

Chia-Hsien Lin

Taiwan

Danhua Lin

China

Hualiang Lin

China

Khin Lin

Myanmar

Candace Lind

Canada

Johanna Lindahl

Sweden

Rebecca Lindberg

Australia

Melissa Lindeman

Australia

Ryan Lindsay

USA

William Lindsley

USA

Jennifer Linkins

Switzerland

Frederick Lipfert

USA

Chang-Pan Liu

Taiwan

Qiyong Liu

China

Xiaodong Liu

USA

Xiaodong Liu

China

Yecai Liu

USA

Youcheng Liu

USA

Siu Hing Lo

UK

Adrian Loerbroks

Germany

Charlotte Löfqvist

Sweden

Carmen Logie

Canada

Carmen Logie

Canada

Terhi Lohela

Finland

Tina Lokke Vie

Norway

Grainne Long

UK

Pat Longmuir

Canada

Claudia Lopes

Brazil

Joao Lopes

Portugal

Maria Jose Lopez

Spain

Nancy Lopez

USA

Noemí López-Perea

Spain

Sarmati Loredana

Italy

Theo Lorenc

UK

Chiara Lorini

Italy

Katie Loth

USA

Olabisi Loto

Nigeria

Lucia Maria Lotrean

Romania

Julia Louw

South Africa

Lise Løvseth

Norway

Markku Löytönen

Finland

David Lubans

Australia

Aaron Lucas

USA

Eliane Lucassen

Netherlands

ALice Lucey

Ireland

Stanley Luchters

Australia

Anna Lugnér

Netherlands

Alessandra Lugo

Italy

Amy Luke

USA

Pisake Lumbiganon

Thailand

Julie Lumeng

USA

Olle Lundberg

Sweden

Katherine Lust

USA

May Nawal Lutfiyya

USA

Deborah Lycett

UK

Ruth Lynfield

USA

Guansheng Ma

China

Qiaoqin Ma

China

Wenjun Ma

China

Ying Ma

China

Doris Ma Fat

Switzerland

Abdellatif Maamri

Morocco

Georgie MacArthur

UK

Alison Macfarlane

UK

Duncan Macfarlane

Hong Kong

Aristides Machado-Rodrigues

Portugal

Mary Mackesy-Amiti

USA

Kelly Mackintosh

UK

Alison Macpherson

Canada

Laura MacRitchie

UK

Jon Macy

USA

Sphiwe Madiba

South Africa

Jeanne Madison

Australia

Ida Madsen

Denmark

John G. Mæland

Norway

Anne Magnus

Australia

Rajendra Maharaj

South Africa

Mohamed Mahfouz

Saudi Arabia

Shehrin Mahmood

Bangladesh

Mary Mahoney

UK

Marina Maior

Brazil

Kwok-Kei Mak

Hong Kong

Kepher Makambi

USA

Jennifer Makin

South Africa

Tomi Mäkinen

Finland

Michael Makowsky

USA

Fredrick Makumbi

Uganda

Ahmad Makuwani

UK

Talia Malagon

Canada

Andre Malbergier

Brazil

Reza Malekzadeh

Iran

Chetna Malhotra

Singapore

Ameer Malik

Pakistan

Asmat Malik

Pakistan

George Mammen

Canada

Kedar Manandhar

Nepal

Samuel Manda

South Africa

Jornt Mandemakers

Netherlands

Ahmed Mandil

Saudi Arabia

Jayanthi Maniam

Azerbaijan

Darrel Manitowabi

Canada

Jonathan Mann

Israel

Alice Mannocci

Italy

Weerawat Manosuthi

Thailand

David Manton

Australia

Lorenzo Mantovani

Italy

Lamberto Manzoli

Italy

Limin Mao

Australia

Myfanwy Maple

Australia

Louise Maple-Brown

Australia

Isabelle Marc

Canada

Ulrich Marcus

Germany

Alessandra Marengoni

Italy

Makino Mariko

Japan

Miguel Mariscal-Arcas

Spain

Julia Marley

Australia

Srinivas Marmamula

India

Manuela Marron

Germany

Gaetano Marrone

Sweden

Brandon Marshall

USA

Amelia Marti

Spain

Kari-Pekka Martimo

Finland

Karen Martin

Australia

Lynne Martin

Australia

Domenico Martinelli

Italy

Priscilla Martinez

USA

Cristina Martinez Martinez

Spain

Alejandro-Cesar Martinez-Baena

Spain

José MIguel Martínez-González

Spain

Sergio Martinez-Hervas

Spain

Vicente Martinez-Vizcaino

Spain

Clarice Martins

Portugal

Duncan Maru

USA

Koutatsu Maruyama

Japan

Alan Maryon-Davis

UK

Paolo Mascagni

Italy

Saba Masho

USA

Saidur Mashreky

Bangladesh

Michael Mason

USA

Mohd Masood

Malaysia

Sabrina Masotti

Italy

Gholamreza Masoumi

Iran

Veronique Massari

France

Peter Massey

Australia

Giuseppe Mastrangelo

Italy

Flora Matheson

Canada

Catherine Mathews

South Africa

Erin Mathieu

Australia

Charles Matthews

USA

Christine Mattson

USA

Pallab Maulik

India

Margaret May

UK

Lindsay Mayberry

USA

Roy William Mayega

Uganda

Miguel Angel Mayer

Spain

Maria Maynard

UK

Marie-Helene Mayrand

Canada

Darren Mays

USA

Sigal Mazor

Israel

Artur Mazur

Poland

Mario Mazzocchi

Italy

Luiz Edmundo Mazzoleni

Brazil

Catherine Mbagaya

Kenya

Blessing Mberu

Kenya

Leonard Mboera

Tanzania

Deborah McCahon

UK

Jim McCambridge

UK

Janet McCarten

USA

Linda McCauley

USA

Joanna McClintock

New Zealand

Scott McCoombe

Australia

Gavin McCormack

Canada

Sandra McCoy

USA

Linda L McCreary

USA

Ted McDonald

Canada

Jim McDonell

USA

Lotus McDougal

USA

Willi McFarland

USA

Chris McGowin

USA

Donovan McGrowder

Jamaica

karma McKelvey

USA

Audrey Mckinlay

New Zealand

Lyle McKinnon

South Africa

James McLay

UK

Kristen McLean

USA

Lucy McLellan

UK

Christopher Mcleod

Canada

David McLoughlin

UK

Robert McMillen

USA

Rebekah McNaughton

UK

Jessica McNeil

Canada

Andrea McNeilly

UK

Anna McNulty

Australia

Amy McQueen

USA

Lorraine McSweeney

UK

Rita McWilliams

USA

Nicolas Meda

Burkina Faso

Kaye Mehta

Australia

Changlin Mei

China

Wubegzier Mekonnen

Ethiopia

Alemayehu Mekonnen Lemma

Ethiopia

Marisa Mena

Spain

Gwenn Menvielle

France

Nicolas Menzies

USA

Kim Meredith-Jones

New Zealand

Dan Merenstein

USA

Cesar Merino

Peru

Michael Merten

USA

Elke Mertens

Germany

Frédéric Mertens

Brazil

Nebiyu Mesfin

Ethiopia

Giuseppe Alessio Messano

Italy

Karen Messing

Canada

Brad Metcalf

UK

Hirohito Metoki

Japan

David Metz

UK

Liesbeth E. Meuwissen

Netherlands

Christian Meyer

Germany

Katie Meyer

USA

Ursina Meyer

Netherlands

Sayoki Mfinanga

Tanzania

Tomasz Miazgowski

Poland

Charles Michelo

Zambia

Nathalie Michels

Belgium

Susan Michie

UK

Kristien Michielsen

Belgium

Luca Miele

Italy

Richard Mihigo

Congo

Jose Mill

Brazil

Lynne Millar

Australia

Brenda A Miller

USA

Brian Miller

UK

Michelle Miller

Australia

Rachel Millstein

USA

Karen Milton

UK

Jennifer Mindell

UK

Barbara Mintzes

Canada

Gita Mishra

Australia

Sharmistha Mishra

Canada

Josef Mitas

Czech Republic

Jordan Mitchell

USA

Richard Mitchell

UK

Steven Mitchell

Canada

Murthy Mittinty

Australia

Kyoko Miura

Australia

Fumihiro Mizokami

Japan

Kristin Mmari

USA

Tirelo Modie-Moroka

Botswana

Gamal Mohamed Ali

Sudan

Mohamed Izham Mohamed Ibrahim

Malaysia

Ghorbanali Mohammadi

Iran

Pavitra Mohan

India

Manoj Mohanan

USA

Sigrid M. Mohnen

Netherlands

Trine Moholdt

Norway

Eiman Mokaddas

Kuwait

Zitha Mokomane

South Africa

Anu Molarius

Sweden

Michal Molcho

Ireland

Nicolas Molinari

France

Mitike Molla

Ethiopia

Liesbeth Mollema

Netherlands

Anders Moller

Sweden

Janne Møller-Stray

Norway

Ana Moncayo

Ecuador

Elisabeth Monnet

France

Joseph Monsonego

France

Isabel Montero

Spain

Catherine Montgomery

UK

Antonio Montresor

Switzerland

John Mooney

UK

Spencer Moore

Canada

Zohar Mor

Israel

Allisyn Moran

USA

Marina Moreira

Brazil

Angelo Moretto

Italy

Miriam Morey

USA

Alison Morgan

Australia

Gemma Morgan

UK

Daniel Morris

USA

Michael Morris

USA

Rory Morrison

UK

Bente Morseth

Norway

S Mohammad J. Mortazavi

Iran

Shlomo Mor-Yosef

Israel

Paola Andrea Mosquera

Colombia

Jorge Mota

Portugal

Varvara Mouchtouri

Greece

Sandra Mounier-Jack

UK

Monique Mourits

Netherlands

Mourad Moursi

USA

Heta Moustgaard

Finland

Francois Moutou

France

Cheryl Moyer

USA

Dariush Mozaffarian

USA

Michael Msall

USA

Gerry Mshana

Tanzania

Stephen Mshana

Tanzania

Mi Msq

China

Kelias Msyamboza

Malawi

Rebecca Muckelbauer

Germany

Aart Nicolaas Mudde

Netherlands

Peter Muennig

USA

Nazeem Muhajarine

Canada

Khitam Muhsen

USA

Arnab Mukherjea

USA

Fidele Kanyimbu Mukinda

South Africa

Paolo Mulatti

Italy

Francis Mulekya Bwambale

Uganda

Afework Mulugeta

Ethiopia

Kevin Mulvey

USA

Yoshitaka Murakami

Japan

Hiroshi Murayama

USA

Adrianna Murphy

UK

James Murphy

USA

Niamh Murphy

Ireland

Christophe Murrill

USA

Maurice Musheke

Zambia

Charles Musselwhite

UK

Charles Musselwhite

UK

Wilkinson Mutahi

Kenya

Francis Mutuku

Kenya

Michael Myers

USA

Vicki Myers

Israel

Arne Kristian Myhre

Norway

Karen Myllynen Webb

Zimbabwe

Julie Mytton

UK

Jean Nachega

USA

Balaji Naik

India

Harish Nair

UK

Maya Nair

USA

Visalini Nair-Shalliker

Australia

Tomoko Nakagami

Japan

Hiroyuki Nakamura

Japan

Mieko Nakamura

Japan

Yuko Nakao

Japan

Gertrude Nakigozi

Uganda

Hairong Nan

Hong Kong

Jolly Nankunda

Uganda

Kulaya Narksawat

Thailand

Zaheer Nasar

UK

Kumari Navaratne

Sri Lanka

Raúl Navarro

Spain

Vincent Navarro

USA

Ana Navas-Acien

USA

Bakhao Ndiaye

France

Abraham Ndiwane

USA

Lee Nedkoff

Australia

Corina Negrescu

USA

Eric Nehl

USA

Torsten Neilands

USA

Michael Nelson

USA

Julianna Nemeth

USA

Tooru Nemoto

USA

Maike Neuhaus

Australia

Dorine Neveu

France

Karl New

UK

Dorothy Newbury-Birch

UK

Tirang R. Neyestani

Iran

Chirk Jenn Ng

Malaysia

Mweete Nglazi

South Africa

Anh Ngo

Viet Nam

Anthony Ngugi

Kenya

Mimmie Claudine Ngum Chi Watts

Australia

Quoc Nguyen

Viet Nam

Thanh Nguyen

Cameroon

Charles Nhan

Canada

Bertha Nhlema-Simwaka

Malawi

Virginie Nicaise

France

Gordon Nichols

Sweden

Nathan Nickel

Canada

Theresa Nicklas

USA

Anne Niec

Canada

Anni Brit Sternhagen Nielsen

Denmark

Linda Nielsen

USA

Martin Lindhardt Nielsen

Denmark

Steffen Niemann

Switzerland

Christopher Niemiec

USA

Albert Nienhaus

Germany

Rathi Nilam

India

Peter Nilsson

Sweden

Feng Ning

Finland

Bernard Njau

Tanzania

Peter Njuho

South Africa

John Nkengasong

USA

Chimeremma Nnadi

USA

Rosekeila Nomelini

Brazil

Astrid Nooyens

Netherlands

Brian Nordmann

USA

Paul Norman

UK

Alexandra Norris

South Africa

Bachok Norsa’adah

Malaysia

Kerri Nottage

USA

Thomas Novotny

USA

Mary Patricia Nowalk

USA

Krzysztof Nowosielski

Poland

Jim Nuovo

USA

Sabina Nuti

Italy

Egil Nygaard

Norway

Carin Nyman

Sweden

Carla Makhlouf Obermeyer

Lebanon

Ursula Oberst

Spain

Ekwaro Obuku

Uganda

Ricardo Ocaña-Riola

Spain

Wendy Oddy

Australia

Maurice Odiere

Kenya

Amos Odiit

Rwanda

Beatrice Odongkara Mpora

Uganda

Oluwakemi Odukoya

Nigeria

Olumuyiwa Odusanya

Nigeria

Okechukwu Ogah

Nigeria

Adesola Ogunniyi

Nigeria

Jude Uzoma Ohaeri

Kuwait

Henrik Ohlsson

Sweden

Koichi Ohno

Japan

Heechoul Ohrr

South Korea

Rie Oka

Japan

Jerry Okal

Kenya

Kamolnetr Okanurak

Thailand

Eileen O’keefe

UK

Chizimuzo Okoli

USA

Tuula Oksanen

Finland

Regina Oladokun

Nigeria

Amos Olagunju

USA

Olusegun Olaoye

Nigeria

Tim Olds

Australia

Caroline O’Leary

UK

Melissa Olfert

USA

Lucio Oliveira

Brazil

Lemessa Oljira

Ethiopia

Lise Olsen

Canada

Nanna Olsen

Denmark

Ashley Olson

UK

Bukola Olutola

South Africa

Jim M O’Mahony

Ireland

Nasrin Omidvar

Iran

Sepideh Omidvari

Iran

Folashade Omokhodion

Nigeria

Ciaran O’Neill

Ireland

Koh Jun Ong

UK

Oluwakare Opaneye

USA

Alloys Orago

Kenya

Ciara O’Reilly

USA

Heather Orpana

Canada

Andrea Orsi

Italy

Letizia Orzella

Italy

Katy Osborne

Australia

Kwadwo Osei Bonsu

Malaysia

Esa Osterberg

Finland

Per-Olof Ostergren

Sweden

John Östh

Sweden

Aleck Ostry

Canada

Theresa Osypuk

USA

Atsuhiko Ota

Japan

David Otiashvili

Georgia

Kennedy Otwombe

South Africa

Odile Ouwe Missi Oukem

Niger

Lijing Ouyang

USA

Nina Cecilie Overby

Norway

Simon Øverland

Norway

Christopher Owen

UK

Irene Oyeflaten

Norway

Koyejo Oyerinde

USA

Adewale Oyeyemi

Nigeria

Sevket Murat Özbek

Turkey

Al Ozonoff

USA

Jorma Paavonen

Finland

Rossana Pacheco da Costa Proenca

Brazil

Ian Pagano

USA

Manacy Pai

USA

Soon-Young Paik

South Korea

Anna Palagyi

Australia

Ståle Pallesen

Norway

Enzo Palombo

Australia

Joan Paluzzi

USA

Chen Wei Pan

Singapore

Liping Pan

USA

Xiao-Chuan Pan

China

Donatella Panatto

Italy

Subadra Panchanadeswaran

USA

Massimiliano Panella

Italy

Puspa Raj Pant

Nepal

Nikos Pantazis

Greece

Maria Papapetropoulou

Greece

George Papathanasiou

Greece

Constantina Papoutsakis

Greece

Catherine Paquet

Australia

Dana Paquette

Canada

Song-Yi Park

USA

Alison Parkes

UK

Diana Parra

USA

Adriana Parrella

Australia

Olavi Parssinen

Finland

Mallie Paschall

USA

Andrew Pask

Australia

Sonia Passos

Brazil

Augusto Alberto Pastorelli

Italy

Antonio Pastorino

Brazil

Jaclyn Paterson

Canada

Jayadeep Patra

Canada

Misti Paudel

USA

Lisa Paul

USA

Theo Paulussen

Netherlands

Bernadette Pauly

Canada

Jane Pearson

USA

Jennifer Pearson

USA

Mangesh Pednekar

India

Anna Peeters

Australia

Karl Peltzer

South Africa

Erica Peluso

Brazil

Hong-Juan Peng

China

Linda Penn

UK

Luke Peppone

USA

Jennifer Pereira

Canada

Adriana Perez

USA

Agustí Pérez Foguet

Spain

Armando Perez-Cueto

Denmark

Christopher Perlman

Canada

Gastón Perman

Argentina

Joseph Perriens

Switzerland

Henry Perry

USA

Robert Perry

Switzerland

Malte Persike

Germany

Lars Åke Persson

Sweden

Armando Peruga

Switzerland

Ruth Peters

UK

Sanne A. E. Peters

Netherlands

Velibor Peters

Netherlands

Irene Petritsi Figa Talamanca

Italy

Stefano Petti

Italy

Tahna Pettman

Australia

Eric Pevzner

USA

Lorenzo Pezzoli

Switzerland

Ceri Phillips

UK

Karen Phillips

Canada

Lisa Phillips

UK

Kai-Lit Phua

Malaysia

Andrew Pick

UK

Mike Pickles

UK

Eduardo David Piemonte

Argentina

Zuzanna Pieniak

Belgium

Gustavo Duarte Pimentel

Brazil

Giovanna Pippa

Italy

Sandra Pisaniello

Australia

Sandra Plachta-Danielzik

Germany

Inger Plaisier

Netherlands

Elodie Plan

Sweden

Rebeca Plank

USA

Andrew Pleasant

USA

Paul L. Plener

Germany

Benjamin Gregory Polkinghorne

Australia

Keshia Pollack

USA

Christina Pollard

Australia

Andrea Polonijo

Canada

Evangelos Polychronopoulos

Greece

Sujata Ponappa

USA

Nancy Poole

Canada

Anil Pooran

South Africa

Jalal Poorolajal

Iran

Diana Pope

USA

Frank Popham

UK

Barry Popkin

USA

Chad Porter

USA

Ann Post

Sweden

Maarten Postma

Netherlands

Walker Poston

USA

Anja Potthoff

Germany

Tracy Pottinger

UK

Christine Poulos

USA

Byron Powell

USA

David Powell

New Zealand

Timothy Powell-Jackson

UK

Krishnananda Prabhu

India

Wagner Luiz Prado

Brazil

Arun Pramanik

USA

Rajendra Prasad

India

Shalini Prasad

India

Tapanan Prateepko

Thailand

Aric Prather

USA

Garrett Prestage

Australia

Andy Pringle

UK

Antonio Prista

Mozambique

Marianne Promberger

UK

Nico Pronk

USA

Karin Proper

Netherlands

Andrea Pugliese

Italy

Anna Puig-Ribera

Spain

Celine Pulcini

France

Elaine Puleo

USA

Richard Pulsford

UK

Vivian Pun

Hong Kong

Narayanan Punithavathi

Malaysia

Narain H. Punjabi

Indonesia

Dinko Puntari¿

Croatia

Lorenza Putignani

Italy

Katarina Putnik

Netherlands

Sampsa Puttonen

Finland

Simona Puzelli

Italy

Harith Qazaz

Iraq

Shuli Qu

USA

Sara Quandt

USA

Xavier Quantin

France

Amelie Quesnel-Vallee

Canada

Micheal Quinlan

Australia

Emma Quinn

Australia

Gwendolyn Quinn

USA

Abdulkareem Quwaidhi

UK

Susan Racette

USA

Bahiri Rachid

Morocco

George Rachiotis

Greece

Beth Rachlis

Canada

Gabriel Rada

Chile

Jany Rademakers

Netherlands

Chantal Raherison

France

Ossi Rahkonen

Finland

Sibylle Rahlenbeck

Germany

Sajjad Rahman

Qatar

Kim Raine

Canada

Anita Raj

USA

Pavani Ram

USA

Shreena Ramanathan

India

Subha Ramanathan

Canada

Rohit Ramaswamy

USA

Josep M Ramon

Spain

Lekhraj Rampal

Malaysia

Yogandree Ramsamy

South Africa

Mats Ramstedt

Sweden

Andrew Ramtohul

UK

Aadia Rana

USA

Raymond Randall

UK

Merja Rantakokko

Finland

Johanna Rantanen

Finland

Krishna Rao

India

Selim Rashed

Canada

Mitra Rashidian

Australia

Mitra Rashidian

USA

Mette Rasmussen

Denmark

Kumari Rathnayake

Sri Lanka

Elena Ratschen

UK

Rainer Rauramaa

Finland

Nidhi Ravishankar

Canada

Carola Ray

Finland

Joel Ray

Canada

H. Fisher Raymond

USA

Amanda Rebar

Australia

Mary Redmayne

New Zealand

Albert Reece

Australia

Marina Reeves

Australia

Krishna Regmi

UK

Naghma Rehan

Pakistan

Joan Reibman

USA

Felipe Reichert

Brazil

Sijmen A. Reijneveld

Netherlands

John Reilly

UK

Thom Reilly

USA

Anne Kerstin Reimers

Germany

Richard Reithinger

USA

Robert Remis

Canada

Dallaire Renee

Canada

Geir K. Resaland

Norway

Bharat Rewari

India

Mae Lynn Reyes-Rodriguez

USA

Grace Reynolds

USA

Lucy Reynolds

UK

Arezoo Rezazadeh

Iran

Giovanni Rezza

Italy

Ana Isabel Ribeiro

Portugal

Dominique Ricard

Laos

Walter Ricciardi

Italy

Brian Rice

UK

Justin Richards

UK

Amanda Richardson

USA

Dawn Richardson

USA

Michael Richardson

USA

Marlise Richter

South Africa

Sarah Riddle

USA

Heather Ridolfo

USA

Susan Rifkin

UK

Simon Riley

UK

Lionel Riou França

France

Laetitia Rispel

South Africa

Antonio Rispo

Italy

Jan Risser

USA

Matti Ristola

Finland

Viviana Ritacco

Argentina

Jan Ritchie

Australia

Anna Ritsatakis

Greece

Evangelos Rizos

Greece

Jose Rizzo

Brazil

Debora Rizzuto

Sweden

Katie Robb

UK

Bayard Roberts

UK

Chris Roberts

UK

Kristin Roberts

USA

Robert Roberts

USA

Susan Roberts

USA

Wendy Robertson

UK

Jude Robinson

UK

Suzan Robroek

Netherlands

Miguel Roca

Spain

Corinne Rocca

USA

Petra Rocic

USA

Simone Rodda

Australia

Rachel Rodgers

USA

Fernando Rodríguez-Artalejo

Spain

Isabel Rodriguez-Barraquer

USA

Corne Roelen

Netherlands

Keetie Roelen

UK

Wendy Rogers

Australia

Peter Rogerson

USA

Kamilla Rognmo

Norway

James Rohrer

USA

Roberto Romi

Italy

Shuang Rong

China

Annet Roodenburg

Netherlands

Kieron Rooney

Australia

Martin Roosli

Switzerland

Elisabeth Root

USA

Annina Ropponen

Finland

Rafaela Rosário

Portugal

Leena Roschier

Finland

Laura Rosella

Canada

Michael Ross

USA

Stephan Rossner

Sweden

Berit Rostad

Norway

Matteo Rota

Italy

Francesco Rotella

Italy

Mohammad Rouhani

Iran

Maria Roura

Spain

Matthew Rousu

USA

Abdulrahim Rouzi

Saudi Arabia

Neneh Rowa-Dewar

UK

Alex Rowlands

UK

Gillian Rowlands

UK

Yuhua Ruan

China

Daniela Rubin

USA

Adolfo Rubinstein

Argentina

Guillaume Ruel

Canada

Joseph Rujumba

Uganda

Godfrey Rukundo

Uganda

Rosemary Rushmer

UK

Tom Rutledge

USA

Femke Rutters

Netherlands

Lucie Rychetnik

Australia

Agnieszka Rychwalska

Poland

Rajaram S Potty

India

Soheil Saadat

Iran

Osmo Saarelma

Finland

Charumathi Sabanayagam

Singapore

Erika Sabbath

USA

Keith Sabin

USA

Osman Sabuncuoglu

Turkey

Masood Sadiq

Pakistan

Ayebo Sadoh

Nigeria

Wilson Sadoh

Nigeria

Sharon Sagiv

USA

Debasish Saha

New Zealand

Klas-Goran Sahlen

Sweden

Thomas Saias

France

Pedro F. Saint-Maurice

USA

Maria Saintrain

Brazil

Ruhi Saith

India

Isao Saito

Japan

Naoki Sakane

Japan

Eduardo Salazar-Lindo

Peru

Marcel Salive

USA

Juha Saltevo

Finland

Giovanni Salum

Brazil

Jonathan Samet

USA

Kiyoshi Sanada

Japan

Alexandra Sánchez

Brazil

Kristy Sanderson

Australia

Gurprataap Sandhu

USA

Helene Sandmark

Sweden

Rassamee Sangthong

Thailand

Maria Paula Santos

Portugal

Rute Santos

Portugal

Lincoln Sargeant

UK

Rajiv Sarkar

India

Tytti Sarkeala

Finland

Olga Sarmiento

Colombia

Radhanath Satpathy

India

Travis Saunders

Canada

Marie-Josephe Saurel-Cubizolles

France

Elena Sautkina

UK

Judith Savageau

USA

Amir Sayem

Bangladesh

Carmen Sayon-Orea

Spain

Shariff-Ghazali Sazlina

Malaysia

Gianpaolo Scalia Tomba

Italy

Elaine Scallan

USA

Michael Scanlon

USA

Christian Schaetti

Switzerland

Ingmar Schafer

Germany

Dena Schanzer

Canada

Malgorzata Schlegel-Zawadzka

Poland

Dan Schlesinger

Canada

Anna Schmidt

Germany

Axel J. Schmidt

Switzerland

Manuela Schmidt

Sweden

Wolf-Peter Schmidt

UK

Margaret Schneider

USA

Inga Schnuerer

Germany

Monica Schoch-Spana

USA

Stephanie Schoeppe

Australia

Gerhard Schön

Germany

Anel Schoonees

South Africa

James Schreiber

USA

Helmut Schroder

Spain

Jochen Schubert

Germany

John Schuna

USA

Victor Schwartz

USA

Kent Schwirian

USA

Bridie Scott-Parker

Bahamas

Lori Scott-Sheldon

USA

Domenico Scrutinio

Ireland

André Seabra

Portugal

Jamie Seabrook

Canada

J Paul Seale

USA

Simon Sebire

UK

Roberto Secades-Villa

Spain

Gina Secura

USA

Sanaz Sedaghat

Netherlands

Laurence Seematter

Switzerland

Jan Seghers

Belgium

John Seifert

USA

Akihiro Seita

Jordan

Jorma Seitsamo

Finland

Peter Selby

Canada

Hilary Seligman

USA

Randi Selmer

Norway

Caroline Semaille

France

Sean Semple

UK

Thomas Semrad

USA

Kate Senior

Australia

Suraj Senjam

India

Teresa Senserrick

Australia

Kwonjune Seung

USA

Ayiche Seyoum

Ethiopia

Amir Shafat

Ireland

Nathan Shaffer

Switzerland

Kashif Shafique

UK

Baiju Shah

Canada

Syed Ghulam Sarwar Shah

UK

Jorida Shahinas

Italy

Mirja Mohammad Shahjamal

Bangladesh

Maryam Shahmanesh

UK

Babar Tasneem Shaikh

Pakistan

Bikash Shakya

USA

Akhawri Shankar

India

Aparna Shankar

UK

Kate Shannon

Canada

Eugene Shapiro

USA

Estifanos Biru Shargie

Switzerland

Surya Kant Sharma

India

Linda Sharp

Ireland

Suresh Shastri

India

Souradet Shaw

Canada

Aubrey Sheiham

UK

Carrington Shepherd

Australia

Roya Sherafat Kazemzadeh

USA

Renslow Sherer

USA

Recinda Sherman

USA

Sandra Sherman

USA

Lijie Shi

Germany

Zumin Shi

Australia

Ruth Shim

USA

Pappur Shivakumar

Canada

Carla Shoff

USA

David Shoham

USA

Rosalyn Shute

Australia

Shannon Sibbald

Canada

Alicja Sieminska

Poland

Jose Sierrra

Spain

Inga Dora Sigfusdottir

Iceland

Lara Sigurdardottir

Iceland

Bergdís Björk Sigurjónsdóttir

Sweden

Claudia Sikorski

Germany

Balewgizie Sileshi

Ethiopia

Roberta Siliquini

Italy

Gustavo Silva

Portugal

Camila Silveira

Brazil

Jay Silverman

USA

Laura Sima

USA

Clifford Simango

Zimbabwe

Daudi Simba

Tanzania

David Simmons

UK

Anne-Laure Simonnot

France

Dorien Simons

Belgium

Gunnar Skov Simonsen

Norway

Eleanor Simonsick

USA

Jamie Sims

UK

John Sirard

USA

John Sirard

USA

Fuschia M. Sirois

Canada

William Sischo

USA

Susan Sisson

USA

Freddy Sitas

Australia

Bonnie WM Siu

Hong Kong

Anna Siukola

Finland

Borge Sivertsen

Norway

Seter Siziya

Zambia

Petros Skapinakis

UK

Natalie Skinner

Australia

Alla Skomorovsky

Canada

Martin Slade

USA

Sandy Slater

USA

Menno Slingerland

Netherlands

Sarah Sliwa

USA

Sinne Smed

Denmark

Eline Smit

Netherlands

Ellen Smit

USA

Albert Smith

USA

Bryce Smith

USA

John Smith

UK

Katherine Smith

USA

Kimberley Smith

Canada

Kylie Smith

Australia

Megan Smith

Australia

Ruth Smith

UK

Mary C Smith Fawzi

USA

Roel Smolders

Belgium

Ian Smythe

UK

John Snelgrove

Canada

Wi-Young So

South Korea

Helene L Soberg

Norway

Lubica Sobotova

Slovakia

Nazmul Sohel

Sweden

Kate Soldan

UK

Michael Soljak

UK

Shawn Somerset

Australia

Isolde Sommer

Austria

francesco sopracordevole

Italy

Lars Sörensen

Finland

Maroje Soric

Croatia

Andre Sourander

Finland

Anastasia Soureti

Greece

Joao Paulo Souza

Switzerland

Marcy Souza

USA

Maria Alice Souza de Oliveira Dode

Brazil

Sin Sovann

Cambodia

Ulla Sovio

UK

Kaan Sözmen

Turkey

Martha Sparks

USA

Laura Spauwen

Netherlands

Maria Lucia Specchia

Italy

Ilene Speizer

USA

Brenda Spencer

Switzerland

Sarah Spengler

Germany

Mark Spigt

Netherlands

Frank Spradley

USA

Sandra Springer

USA

Chandrashekhar Sreeramareddy

Nepal

steven stack

USA

Massimo Stafoggia

Italy

Mandy Stahre

USA

Sharon Stancliff

USA

James Stanley

New Zealand

Victoria Staples

UK

Martine Stead

UK

Richard Steckel

USA

Leah Steele

Canada

Robert Steele

UK

Richard Steen

Italy

Alexander J. Stein

USA

Peter Steinmann

Switzerland

Sarit Steinmetz

Israel

Marlene Stenbacka

Sweden

Craig Stephen

Canada

Lena Stephens

Australia

Nathan Stephens

UK

Nardi Steverink

Namibia

Wayne Steward

USA

Christine Stewart

USA

Jane Stewart

UK

Jessica Stewart

Australia

Nelia Patricia Steyn

South Africa

Margaret Stineman

USA

Christiane Stock

Denmark

Emily Stockings

Australia

Jamila Stockman

USA

Barbara Stoecker

USA

Heather Stombaugh

USA

Michelle Stone

Canada

Jessica Storbjörk

Sweden

Ingunn Størksen

Norway

Kristi Storti

USA

Louise St-Pierre

Canada

Renee St-Pierre

Canada

Bjørn Heine Strand

Norway

Mark Strand

USA

Katrine Strandberg-Laren

Denmark

Kathrin Strasser-Weippl

Austria

Babill Stray-Pedersen

Norway

Renate Strehlau

South Africa

Silvia Stringhini

Switzerland

Claudia Strugnell

Australia

Patricia Struthers

South Africa

Heather Stuart

Canada

Andreas Stuck

Switzerland

Pedro Suarez

USA

Eva Suarthana

Canada

Malavika Subramanyam

India

Pavel Suchanek

Czech Republic

Parminder Suchdev

USA

L Suzanne Suggs

Switzerland

Koshu Sugisaki

Japan

Kadri Suija

Finland

Michael Sullivan

USA

Patrick Sullivan

USA

Markku Pertti Tapio Sumanen

Finland

Walton Sumner

USA

Linas Sumskas

Lithuania

Neisha Sundaram

Switzerland

Karin Sundström

Sweden

Saradha Suresh

India

Jeremy Sussman

USA

Marie Suzan-Monti

France

Etsuji Suzuki

Japan

Chalida Svastisalee

Denmark

Larry Svenson

Canada

Madeleine Svensson

Sweden

Monica Swahn

USA

Emmanuel Swai

Tanzania

Soumya Swaminathan

India

Dallas Swendeman

USA

Masila Syengo

Kenya

Karolina Szerencsi

Netherlands

George Szmukler

UK

Dorota Szostak-Wegierek

Poland

Simon Szreter

UK

Birkneh Tadesse

Ethiopia

Tadese Ejigu Tafere

Ethiopia

Silvio Tafuri

Italy

Harry Tagbor

Ghana

Richard Tahtinen

Iceland

Daisuke Takagi

Japan

Masaya Takahashi

Japan

Simbarashe Takuva

South Africa

Marina Taloyan

Sweden

Clarence Tam

UK

Hiko Tamashiro

Japan

Darrell Tan

Canada

Stephanie Tanamas

Australia

Pooja Tandon

USA

Elisabetta Tanzi

Italy

Beatriz Tavares

Brazil

Christopher Taylor

USA

Crystal Taylor

USA

Douglas Taylor

USA

Lee Taylor

Australia

Mostafa Taymour

Egypt

Yibeltal Tebekaw

Ethiopia

Abebe Kebede Teferi

Ethiopia

Fasil Tekola-Ayele

USA

Shirley Telles

India

Henriëtte ter Waarbeek

Netherlands

Megan Teychenne

Australia

Duong Thanh

Viet Nam

Dag Steinar Thelle

Norway

Maria Theodoridou

Greece

Tores Theorell

Sweden

Grace Thoithi

Kenya

Audrey Alforque Thomas

Ireland

David Thomas

Australia

Roger Thomas

Canada

Ronald Thomas

USA

Sandra Thomas

USA

Sara Thomée

Sweden

Bruce Thompson

Australia

Kara Thompson

Canada

Jo Thompson Coon

UK

Sallie Thoreson

USA

Ingibjörg Thorisdottir

Iceland

Joanna Thorn

UK

Louise Thornton

Australia

Anne Marie Thow

Australia

Wilfreda E. Thurston

Canada

Benedikt Till

Austria

Lesley Tilson

Ireland

Tananze Tinati

UK

Matias Tisi Baña

Argentina

Jim Todd

Tanzania

Savvas Tokmakidis

Greece

Mimmi Tolvanen

Finland

Ildiko Tombor

UK

Seng Fah Tong

Malaysia

Thelma Toni-Uebari

UK

Liv Elin Torheim

Norway

HIroyuki Torisu

Japan

José Torres da Costa

Portugal

Evelyne Touchette

Canada

Alberto Tozzi

Italy

Thach Tran

Viet Nam

Vianney Tricou

Central African Republic

Dominique Tripodi

France

Alexander Tsai

USA

Jack Tsai

USA

Rebecca Tsai

USA

Haritini Tsangari

Cyprus

Sandy Tubeuf

UK

Carrie Tudor

USA

Olivia Tulloch

UK

Mark Tully

UK

Jingsheng Tuo

USA

Philippe Tuppin

France

Karen Turner

Australia

Katy Turner

UK

Lindsey Turner

USA

Lisa Tussing-Humphreys

USA

Torill Helene Tveito

Norway

Kimberly Tyler

USA

Tore Tynes

Norway

Dong-Sheng Tzeng

Taiwan

Konstantinos Tziomalos

Greece

Kingsley Nnanna Ukwaja

Nigeria

Noman ul Haq

Pakistan

Belgin Unal

Turkey

Jessica Unick

USA

Brigid Unim

Italy

Róbert Urbán

Hungary

Karen Urbanoski

Canada

Sorin Ursoniu

Romania

Avita Usfar

Indonesia

Joan Vaccaro

USA

Isabelle Vachier

France

Tone Gretland Valderhaug

Norway

Jara Valtuena

Spain

Mahara Valverde

Mexico

Jan van Amsterdam

Netherlands

Ed van Beeck

Netherlands

Mirjam Elisabeth Johanna van Beelen

Netherlands

Bart van den Borne

Netherlands

Ingrid Viola Francine van den Broek

Netherlands

Jef Van den Ende

Belgium

Jaap van den Heuvel

Netherlands

Edith van den Hooven

Netherlands

Anke van der Kwaak

Netherlands

Daphne van der A

Netherlands

Leontien van der Knaap

Netherlands

Zephne M. van der Spuy

South Africa

Marieke J. van der Werf

Sweden

Dane Van Domelen

USA

rogier van doorn

Viet Nam

Saskia van Dorsselaer

Netherlands

Guido Van Hal

Belgium

Geneviève van Liere

Netherlands

Wendy Van Lippevelde

Belgium

Yvonne van Mourik

Netherlands

Rudolph Leon van Niekerk

South Africa

Sandra Helena van Oostrom

Netherlands

Herman Van Oyen

Belgium

Mireille van Poppel

Netherlands

Joop van Raaij

Netherlands

Jos van Roosmalen

Netherlands

Karin van Rosmalen-Nooijens

Netherlands

Ronan Van Rossem

Belgium

Esther van Sluijs

UK

Irene van Staveren

Netherlands

Berna van Wendel de Joode

Costa Rica

Peter Vanable

USA

Kerry Vander Ploeg

Canada

Kristin Vanderende

USA

Patrik Jules Maria Vankrunkelsven

Belgium

Veerle Vanlerberghe

Belgium

Jacques Vanobbergen

Belgium

Jeffrey VanWormer

USA

Maria Inês Varela-Silva

UK

Juan Vasconez

Ecuador

Eleftheria Vasileiadou

Netherlands

Clemente Vásquez

Mexico

Prin Vathesatogkit

Thailand

Cathy Vaughan

Australia

Amber Vaughn

USA

Fernando Vázquez

Spain

Jaap Veen

Netherlands

Tener Veenema

USA

Arto Vehviläinen

Finland

Juha Veijola

Finland

Jeremy Veillard

Canada

Raman Velayudhan

Switzerland

Lydian Veldhuis

Netherlands

Maria Assunta Veneziano

Italy

Maria Amelia Veras

Brazil

Marie-Noël Vercambre

France

Petra Verdonk

Netherlands

Stephane Verguet

USA

Nick Verhaeghe

Belgium

Kirsten Verkooijen

Netherlands

Marina Verlinden

Netherlands

Arpana Verma

UK

Sylvia Vermeulen

Netherlands

Sten Vermund

USA

Suzanne Verver

Netherlands

Paul Veugelers

Canada

Nicolas Veziris

France

Francesca Viazzi

Italy

Alejandro Videla

Argentina

Damon Vidrine

USA

Alessandra Viganò

Italy

Luz Maria Vilca Yengle

Spain

Raquel Villegas

USA

Paul Villeneuve

Canada

Sylvie Vincent-Höper

Germany

Christopher Vinnard

USA

Yana Vinogradova

UK

Jesus Vioque

Spain

Marianna Virtanen

Finland

Javier Virués-Ortega

Canada

Fehmida Visnegarwala

India

Frank Visseren

Netherlands

Gopalan Viswanathan

India

Philip Vita

Australia

Francesco Vitale

Italy

Barbara Vizmanos

Mexico

Hristina Vlajinac

Serbia

Andrew Voetsch

USA

Nicole Vogelzangs

Netherlands

Albert Vollaard

Netherlands

Ellen Volpe

USA

Mikaela von Bonsdorff

Finland

Rüdiger von Kries

Germany

Kuku Voyi

South Africa

Bernard Vrijens

Belgium

Bao Vu

Viet Nam

Bea Vuylsteke

Belgium

Joseph Vyankandondera

Australia

Krishna Vyncke

Belgium

Dale Wagner

USA

Gabriela Wagner

Brazil

Kolawole Wahab

Nigeria

Mark Wahlqvist

Taiwan

Ichiro Wakabayashi

Japan

Kristin Wall

USA

Lorraine Wallace

USA

Thorne Wallman

Sweden

Helen Walls

Australia

Ken Wallston

USA

David Walsh

UK

Thomas Wan

USA

Handan Wand

Australia

JianLi Wang

Canada

Jianming Wang

China

Jie Jin Wang

Australia

Min-Jung Wang

Australia

Peiyu Wang

China

Shigong Wang

China

Weibing Wang

China

Yan Wang

USA

Zhihong Wang

China

Jeni Warburton

Australia

Catharine Ward Thompson

UK

Jonathan Warren

UK

Petra Warschburger

Germany

Reynold Washington

India

Ian Watt

UK

Helen Weatherly

UK

David Webb

UK

Mary Beth Weber

USA

Bonnie Webster

UK

Premila Webster

UK

Chongyi Wei

USA

Xiaolin Wei

China

Catherine Weil-Olivier

France

Stevan Weine

USA

Heide Weishaar

UK

Barry Weiss

USA

Helen Weiss

UK

Elizabeth Weist

USA

Renata Welc-Faleciak

Poland

Robert Wellman

USA

Cherie Wells

Australia

Margaret Wells

USA

Xiaozhong Wen

USA

Xiaozhong Wen

USA

Thomas Wenzel

Austria

Guilherme Werneck

Brazil

Bo Werner

Sweden

Keon West

UK

Nelli Westercamp

USA

Peter J.M. Westerholm

Sweden

Catherine Wetmore

USA

Rosa Whalen

UK

Benedict Wheeler

UK

Jennifer White

Australia

Katherine White

Australia

Melicia Whitt-Glover

USA

Hilton Whittle

UK

Kremlin Wickramasinghe

UK

Kurt Widhalm

Austria

Amelie Wiedemann

Germany

Mark Wieland

USA

Daniel Wight

UK

Linda Wijlaars

UK

Stefan Wiktor

Switzerland

Joellen Wilbur

USA

Anna Wilkinson

USA

Ingrid Willaing

Denmark

Anna Williams

Australia

Emily Williams

UK

Lauren Williams

Australia

Andrew Willis

UK

Paul Willis

UK

Nevin Wilson

India

Sarah Wilson

Canada

Carl Johan Wingren

Sweden

Sandra Winter

USA

Carl Heinz Wirsing von Koenig

Germany

Marilyn Wise

Australia

Larry Wissow

USA

Jerome Wittwer

France

Pia Wohland

UK

Luke Wolfenden

Australia

Mekitie Wondafrash

Ethiopia

Yirga Wondemnhe

Ethiopia

Fiona Wong

Hong Kong

Frank Wong

Uzbekistan

Paul W.C. Wong

Hong Kong

T.Y. Wong

Singapore

William W. L. Wong

Canada

Lesley Wood

South Africa

Sara Wood

UK

Michael Woodford

USA

Sarah Woodhall

UK

Cynthia Woodsong

South Africa

Helen Worthington

UK

Adugna Woyessa

Ethiopia

Jim Wright

UK

Marcie Wright

USA

Alison Wringe

UK

Fei-Ling Wu

Taiwan

Winfred Wu

USA

Sara Wuehler

Ethiopia

Gwenllian Wynne-Jones

UK

Karen Wynter

Australia

Lin Xiao

China

Shifu Xiao

China

Xiao-Peng Xiong

USA

Fei Xu

China

Kapil Yadav

India

Walelegn Worku Yallew

Ethiopia

Taiki Yamaji

Japan

Hong Yan

China

Baohui Yang

Australia

Joyce Yang

USA

Lin Yang

Hong Kong

Isaac Kofi Yankson

Ghana

Sylvia Yano

Bouvet Island

Alfred Yawson

Ghana

Alison Yaxley

Australia

Mahbuba Yeasmin

Australia

Kojo Yeboah-Antwi

USA

Ju-Yu Yen

Taiwan

Sern Wei Yeoh

Australia

Jin-Sang Yoon

South Korea

April Young

USA

David Young

Australia

Dongbao Yu

Switzerland

Hongmei Yu

China

Jinming Yu

China

Ruby Yu

Hong Kong

Shumei Yun

USA

Fakir Yunus

Bangladesh

Oyindamola Yusuf

Nigeria

Apolinaras Zaborskis

Lithuania

Emilia Zainal Abidin

UK

Roger Zapata

Peru

Valeria Zavan

Italy

Philippe Zawieja

France

Ayman Zayed

Jordan

Maria-Cecilia Zea

USA

Hajo Zeeb

Germany

Luiz Zeferino

Brazil

Donald Zeigler

USA

Jean-Pierre Zellweger

Switzerland

Elsa Zerbini

Argentina

Jianzhen Zhang

Australia

Junhua Zhang

China

Kai Zhang

USA

Liying Zhang

USA

Yaobi Zhang

Senegal

Yonghong Zhang

China

Dong Zhao

China

Lina Zhao

USA

Yuejen Zhao

Australia

Chen Zhen

USA

Pinpin Zheng

China

Yong-Tang Zheng

China

Youfei Zheng

China

Liang Zhou

China

Chnuyan Zhu

China

Cathleen Zick

USA

Claire Zizza

USA

Denis Zmirou-Navier

France

Peter Zock

Netherlands

Heinz Zotter

Austria

Sa’ed Zyoud

Palestinian Territory

## Pre-publication history

The pre-publication history for this paper can be accessed here:

http://www.biomedcentral.com/1471-2458/14/81/prepub

